# Respiratory kinematic and airflow differences between reflex and voluntary cough in healthy young adults

**DOI:** 10.3389/fphys.2015.00284

**Published:** 2015-10-09

**Authors:** Alexandra E. Brandimore, Michelle S. Troche, Jessica E. Huber, Karen W. Hegland

**Affiliations:** ^1^Department of Speech, Language, and Hearing Sciences, University of FloridaGainesville, FL, USA; ^2^Malcom Randall VA Medical Center, Brain Rehabilitation Research CenterGainesville, FL, USA; ^3^Department of Biobehavioral Sciences, Teachers College Columbia UniversityNew York, NY, USA; ^4^Department of Speech, Language, and Hearing Sciences, Purdue UniversityWest Lafayette, IN, USA

**Keywords:** cough, reflex, voluntary, respiratory kinematics, healthy adults

## Abstract

**Background:** Cough is a defensive behavior that can be initiated in response to a stimulus in the airway (reflexively), or on command (voluntarily). There is evidence to suggest that physiological differences exist between reflex and voluntary cough; however, the output (mechanistic and airflow) differences between the cough types are not fully understood. Therefore, the aims of this study were to determine the lung volume, respiratory kinematic, and airflow differences between reflex and voluntary cough in healthy young adults.

**Methods:** Twenty-five participants (14 female; 18–29 years) were recruited for this study. Participants were evaluated using respiratory inductance plethysmography calibrated with spirometry. Experimental procedures included: (1) respiratory calibration, (2) three voluntary sequential cough trials, and (3) three reflex cough trials induced with 200 μM capsaicin.

**Results:** Lung volume initiation (LVI; *p* = 0.003) and lung volume excursion (LVE; *p* < 0.001) were significantly greater for voluntary cough compared to reflex cough. The rib cage and abdomen significantly influenced LVI for voluntary cough (*p* < 0.001); however, only the rib cage significantly impacted LVI for reflex cough (*p* < 0.001). LVI significantly influenced peak expiratory flow rate (PEFR) for voluntary cough (*p* = 0.029), but not reflex cough (*p* = 0.610).

**Discussion:** Production of a reflex cough results in significant mechanistic and airflow differences compared to voluntary cough. These findings suggest that detection of a tussigenic stimulus modifies motor aspects of the reflex cough behavior. Further understanding of the differences between reflex and voluntary cough in older adults and in persons with dystussia (cough dysfunction) will be essential to facilitate the development of successful cough treatment paradigms.

## Introduction

Cough is a sensorimotor airway protective mechanism that functions to expel foreign or endogenously produced material from the lower airways (Loudon and Shaw, 1967; Harris and Lawson, 1968; Macklem, 1974). There are two distinct types of cough that have been discussed in the literature: voluntary cough and reflex cough. Voluntary cough is initiated on command (Lee et al., 2002; Eccles, 2009; Hegland et al., 2012; Mazzone et al., 2013), whereas reflex cough is initiated secondary to the stimulation of peripheral sensory receptors (Mazzone, 2005; Widdicombe and Singh, 2006; Canning and Chou, 2009). It is well-established that there are differences in the central neural control of the cough types (Lee et al., 2002; Mazzone et al., 2007, 2013; Eccles, 2009; Farrell et al., 2012; Troche et al., 2014a); however, the peripheral structures comprising the final common pathway for task execution are equivalent for both voluntary and reflex cough (Lasserson et al., 2006; Magni et al., 2011). Although the neural differences have been relatively well studied, the airflow and kinematic differences between reflex and voluntary cough are not as well understood, leaving a significant gap in our understanding of the important differences (or similarities) between the two cough types. This is important because reflex and voluntary cough deficits exist in patient populations; yet, it remains unclear how the mechanisms of both cough types differ in healthy populations without the presence of age- or disease-related deficits. Therefore, the goal of this study was to determine the respiratory kinematic, lung volume, and airflow differences between reflex and voluntary cough in healthy young adults.

**Figure 1:**
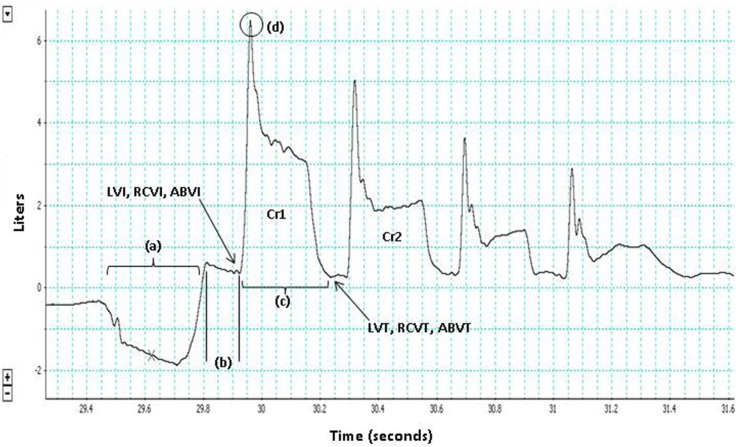
**The three phases of cough are depicted for the first cough in this voluntary cough epoch (Cr1): (a) inspiration, (b) compression, (c) expulsion**. Lung volume initiation (LVI), rib cage volume initiation (RCVI), and abdominal volume initiation (ABVI) are measured at the termination of the compression phase prior to each cough in the cough epoch. Lung volume termination (LVT), rib cage volume termination (RCVT), and abdominal volume termination (ABVT) are measured at the termination of the expulsive phase of each cough in the cough epoch. Peak expiratory flow rate (PEFR) (d); the second cough response in the epoch (Cr2).

Both reflex and voluntary cough are comprised of three phases (inspiration, compression, and expulsion), that temporally sequenced result in dynamic compression of the airways (Ross et al., 1955; Harris and Lawson, 1968; Macklem, 1974; Widdicombe and Singh, 2006; Davenport, 2009; Figure 1). Despite the similarities in the peripheral phases of reflex and voluntary cough, researchers have identified some physiological differences between the cough types (Lasserson et al., 2006; Lavorini et al., 2007). At the muscular level, researchers have shown differences in the functional organization and coordination of muscular activity between reflex and voluntary cough (Lasserson et al., 2006). For example, a study by Lasserson et al. (2006) found that mean abdominal expiratory muscle electromyography (EMG) activity for reflex cough was greater, with shorter burst duration, compared to voluntary coughs with equivalent airflow rates. Additionally, the authors reported that reflex cough resulted in simultaneous onset of expiratory and accessory inspiratory muscle (i.e., trapezius, pectoralis major, deltoid, latissimus dorsi) EMG activity, while voluntary cough resulted in a more graded, coordinated increase in expiratory and accessory inspiratory muscle EMG activity.

The physiology of cough has also been studied by evaluating the kinematic characteristics of the respiratory apparatus (Lanini et al., 2007; Smith et al., 2012). Given that lung volume changes as a function of respiratory muscle contraction within the chest wall (comprised of the rib cage, abdomen, and diaphragm; Hoit, 1995), respiratory kinematic techniques, including body surface plethysmography, provide useful, non-invasive methods that measure the circumferential changes of the rib cage and abdomen (Konno and Mead, 1967; Hixon, 1973; Hixon et al., 1976). This kinematic data identifies how lung volume change is partitioned among the parts of the respiratory apparatus, and highlights the strategies people employ to perform functional tasks (Hixon, 1973; Huber et al., 2005). Additionally, these measures can be directly related to cough airflow parameters if simultaneously assessed using spirometry. Smith et al. (2012) completed the first and only study evaluating the respiratory kinematics of reflex and voluntary cough in healthy young adults. They found that participants were able to achieve higher targeted operating volumes [similar to lung volume initiation (LVI)] prior to voluntary cough compared to reflex cough induced with citric acid (Smith et al., 2012). The authors also found that operating volume was the greatest determinant of lung volume expelled during both reflex and voluntary cough tasks, and peak expiratory airflow rate (PEFR) achieved during voluntary cough (Smith et al., 2012). However, measures of airflow were not obtained from reflex coughs in that study which limited the comparisons that can be made between the mechanisms of voluntary and reflex cough.

The goal of this study was to identify lung volume, respiratory kinematic, and airflow parameters that differ during reflex and voluntary cough in healthy young adults. Based on previous studies (Lanini et al., 2007, 2008; Smith et al., 2012), we hypothesized that voluntary cough would result in greater LVI and lung volume excursion (LVE), with lower lung volume termination (LVT), compared to reflex cough. Additionally we hypothesized that rib cage and abdominal displacement would significantly contribute to LVI for both reflex and voluntary cough. The secondary aim of this study was to determine the effects of lung volume on PEFR for reflex and voluntary cough. Based on previous research (Smith et al., 2012), we hypothesized that LVI would significantly influence PEFR for both reflex and voluntary cough.

## Methods

The Institutional Review Board (IRB) at the University of Florida approved all study procedures, and all participants provided verbal and written informed consent. We recruited 25 young adults (14 female; 18–29 years). Participants reported no history of smoking, respiratory disease (including asthma), neurological disease, recent chest infection in the last 5 weeks, or capsaicin allergy. Participants had normal forced vital capacity (VC) defined as >80% of expected values based on age, sex, height, and weight (Knudson et al., 1983; Miller et al., 1986, 2005). Participant demographic information is in Table 1.

**Table 1:** **Participant demographics**.

**Participant demographics**	**Means ± Standard deviations (SD)**
Participants	*n* = 25
Age (years)	*F* = 23.3 (*SD* = 3.60); *M* = 23.4 (*SD* = 3.20) (Range: 18–29)
Sex	*F* = 14; *M* = 11
Weight (kg)	*F* = 60.95 (*SD* = 9.50); *M* = 79.05 (*SD* = 1.20)
Height (inches)	*F* = 65.7 (*SD* = 2.31); *M* = 71.1 (*SD* = 1.83)
BMI (kg/m)	*F* = 21.75 (*SD* = 2.60); *M* = 24.18 (*SD* = 2.69)

*F, Female; M, male; n, number of participants; SD, standard deviation; BMI, body mass index; kg, kilograms; kg/m, kilograms per meter*.

### Equipment

#### Respiratory kinematics

Respiratory inductance plethysmography via the Respitrace system (Ambulatory Monitoring, Inc., Ardsley, NY) was used to transduce respiratory movements and associated changes in lung volume during cough production. To track rib cage movement, one cotton elastic band was placed circumferentially under the axilla, and to track abdominal movement, one band was placed around the abdomen with the top of the band at the level of the umbilicus, below the last rib. Respiratory data was collected during all study procedures, including during calibration procedures [i.e., rest breathing, VC, and abdomen-in (AB-in) and abdomen-out (AB-out) maneuvers; Hoit and Hixon, 1987], voluntary cough tasks, and reflex cough tasks.

#### Airflow

Airflow measures were collected using a face mask held securely over each participant's nose and mouth. The face mask was coupled to a pneumotachograph system that input differential pressure change to a digital spirometer (MLT 1000, ADInstruments, Inc.). The pneumotachograph system had a side delivery port with a one-way inspiratory valve for nebulizer connection. The nebulizer was a DeVilbiss T-piece (DeVilbiss Healthcare) connected to a dosimeter (KoKoDigidoser; Pulmonary Data Services Instrumentation Inc; Louisville, CO.) that delivered a single aerosolized dose of 200 μM capsaicin (cough inducing stimuli) upon detection of an inspired breath with a delivery duration of 2 s. Reflex coughs that occurred within 15 s of capsaicin delivery were identified and counted. The same spirometric apparatus, without capsaicin, was used to assess voluntary cough function. This methodology has been used in previous research studies (e.g., Vovk et al., 2007; Davenport et al., 2009; Hegland et al., 2014) to record cough airflow.

#### Procedures

During data collection, participants were seated with their feet flat on the floor and arms resting on the armrests to minimize movement artifact. For instrument calibration purposes, participants completed a minute of rest breathing, three VC maneuvers to acquire an estimate of the maximal capacity of the lungs and rib cage, and three maximal AB-in and AB-out maneuvers beginning at end-expiratory level (EEL) to acquire an estimate of the maximal capacity of the abdomen (Hoit and Hixon, 1987; Hoit et al., 1989; Huber et al., 2005). For the AB-in maneuver, participants were cued to hold their breath at EEL and to suck their belly in as far as possible. For the AB-out maneuver, participants were cued to hold their breath at EEL and extend their belly out as far as possible. Experimental cough procedures followed calibration procedures, and included: (1) three, voluntary sequential cough trials during which participants were cued to “cough like something went down the wrong pipe,” and (2) three, reflex cough trials induced with 200 μM capsaicin during which participants were instructed to “cough if you need to.” We chose this set of verbal instructions for voluntary and reflex cough based on our research that has shown that the number of coughs within an epoch significantly influences the airflow parameters of the first and second coughs produced (Hegland et al., 2013). Reflex cough testing using 200 μm capsaicin has shown to reliably produce at least two coughs in young, healthy adults (Vovk et al., 2007; Hegland et al., 2013). Thus, for voluntary cough, an instruction that would elicit multiple coughs was utilized to limit the differences between production of the cough types. Participants were provided with a 20 min break in between completion of the voluntary and reflex cough tasks.

The airflow and kinematic data were input to a Power Lab Data Acquisition System (ML870/P ADInstruments), digitized, and recorded to a Dell laptop computer using Chart 7 software (ADInstruments, Inc.). These data were digitized at 2000 Hertz (Hz).

#### Data analysis

Respiratory kinematic signals were low-pass filtered at 50 Hz to remove high frequency noise from the signal. Respiratory kinematic measurements were made using custom algorithms written in Matlab (MathWorks, version 2011b). Lung volume changes reflect combined changes in rib cage and abdomen volumes (Konno and Mead, 1967). Therefore, the sum of the rib cage and abdomen signals were computed and corrected for their respective contributions to lung volume. The spirometer, rib cage, and abdomen signals during the rest breathing task were also used to determine correction factors for the rib cage and abdomen in order to estimate lung volume during the cough tasks. The Moore-Penrose pseudoinverse function was used in Matlab to determine the least errored solution for the correction factors (k_1_ and k_2_). The pseudoinverse function solved for k_1_ and k_2_in the formula: spirometry volume = k_1_(rib cage) + k_2_(abdomen) for each set of data points in the rest breathing task. Estimated lung volume was then computed for each point during the cough tasks using the same formula. The method has been used effectively in previous kinematic investigations (Chadha et al., 1982; Huber et al., 2005; Huber and Spruill, 2008; Stathopoulos et al., 2014).

Kinematic measurements were expressed as a percent of VC, rib cage capacity, or abdominal capacity, relative to EEL. Hence, positive values are above EEL; and negative values are below EEL. For data analyses, EEL was taken as the average of three consecutive minimum values before each task from the lung, rib cage, and abdomen waveforms (Konno and Mead, 1967; Hixon, 1973; Stathopoulos and Sapienza, 1997; Huber et al., 1998, 2005; Huber, 2007; Huber and Spruill, 2008; Wheeler Hegland et al., 2009, 2011; Stathopoulos et al., 2014).

LVI, rib cage volume initiation (RCVI), and abdominal volume initiation (ABVI) were measured at the termination of the compression phase prior to each cough in the cough epoch (Figure 1). LVT was measured at the termination of each cough in the cough epoch (Figure 1). LVE was calculated as the volume at initiation minus the volume at termination (i.e., LVI-LVT). PEFR (l/s) served as the primary measure of airflow in this study and was measured for each cough in a cough epoch (Figure 1).

#### Statistics

Means were computed for each participant for the first two cough responses (Cr1 and Cr2) in each cough epoch across the three trials of each cough task (voluntary and reflex; Figure 1). Two-way repeated measures ANOVAs tested the differences in mean lung volumes by cough type (voluntary vs. reflex) and cough response number (Cr1 vs. Cr2). Sex served as the between-subjects factor to identify potential differences between males and females. Tukey's HSD tests were completed for all interactions that were significant in the ANOVAs. Correlational analyses were used to identify collinear factors. Separate linear regression models were used to determine the contribution of the rib cage and abdomen on LVI, as well as the impact of LVI on PEFR, during the two cough tasks. Alpha level for the ANOVAs, regressions, and Tukey's HSD was *p* < 0.05.

## Results

Of the 25 participants (mean age = 23 years) enrolled in the study, four did not respond to 200 μM capsaicin. Therefore, 21 participants were included in the analyses. Participants produced two or more sequential coughs in both the voluntary and reflex tasks (mean reflex cough response = 3.49; mean voluntary cough response = 3.64), minimizing any effect that number of coughs would have on the results. There was no significant main effect for sex.

**Figure 2:**
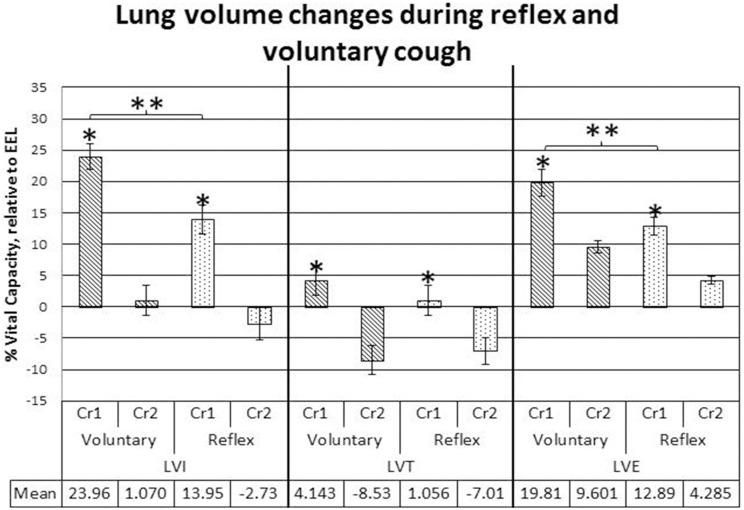
**Lung volume initiation (LVI), termination (LVT), and excursion (LVE) measurements during voluntary and reflex cough: mean percent vital capacity difference relative to end-expiratory level (EEL) for the first and second cough responses in a cough epoch**. Lines show standard errors. The first cough response in an epoch (Cr1); second cough response in an epoch (Cr2); EEL. ^*^Lung volumes for Cr1 were significantly greater than lung volumes for Cr2. ^**^Significant lung volume differences between reflex and voluntary cough (*p* < 0.05).

**Figure 3:**
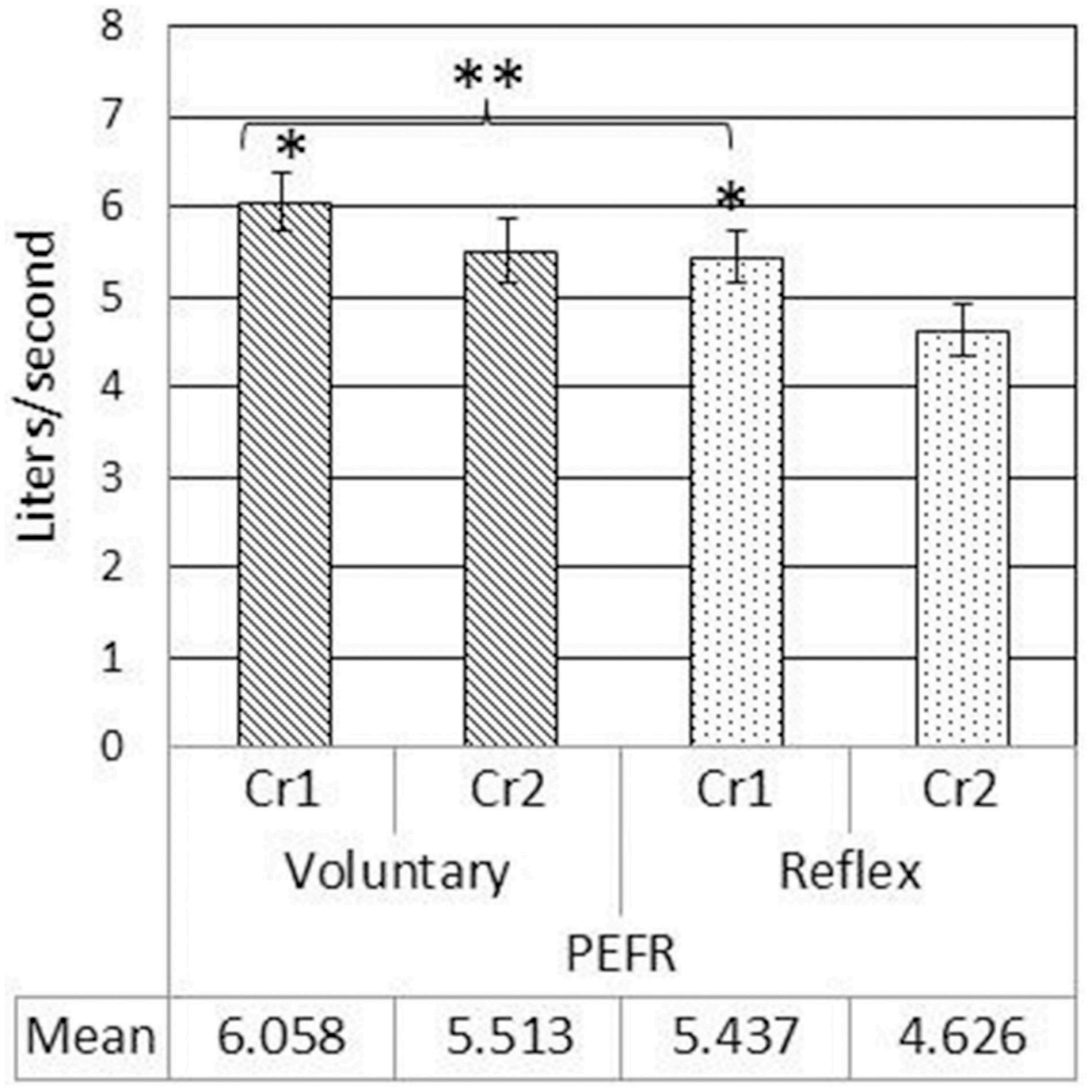
**Mean peak expiratory flow rate (PEFR) measurements during voluntary and reflex cough for the first (Cr1) and second (Cr2) cough responses**. Lines show standard errors. ^*^PEFR for Cr1 was significantly greater than PEFR for Cr2. ^**^Significant PEFR differences between reflex and voluntary cough (*p* < 0.05).

LVI and LVE were significantly greater for voluntary cough compared to reflex cough (Figure 2). Additionally, LVI, LVT, and LVE were significantly greater for Cr1's compared to Cr2's (Figure 2). PEFR was also significantly greater for voluntary cough compared to reflex cough, and greater for Cr1's compared to Cr2's (Figure 3). Table 2 provides a summary of the statistical results.

**Table 2:** **Statistical summary for cough type, and cough response number, and cough type by cough response number interactions**.

**Measures**	**Cough type (voluntary vs. reflex) (1,20)**			**Cough response number (Cr1 vs. Cr2) (1,20)**			**Cough type × cough response number (1,20)**		
	***F***	***n*^2^**	***P***	***F***	***n*^2^**	***P***	***F***	***n*^2^**	***P***
Lung volume initiation (LVI)	11.66	0.37	*P* = 0.003^*^	168.19	0.89	*P* < 0.001^*^	7.61	0.28	*P* = 0.012^*^
Lung volume termination (LVT)	0.13	0.01	*P* = 0.718	319.16	0.94	*P* < 0.001^*^	14.30	0.42	*P* = 0.001^*^
Lung volume excursion (LVE)	25.04	0.56	*P* < 0.001^*^	51.58	0.72	*P* < 0.001^*^	0.33	0.02	*P* = 0.573
Peak expiratory airflow rate (PEFR)	6.93	0.26	*P* = 0.016^*^	23.77	0.54	*P* < 0.001^*^	1.80	0.08	*P* = 0.195

*Degrees of freedom shown in parentheses. F, F ratio; n^2^, partial eta squared; P, level of significance*.

* *Significance at the P < 0.05 level*.

Significant two-way cough type by cough response number interaction effects were found for LVI and LVT (Figure 2). Exploration of the interactions revealed significantly greater LVI for voluntary Cr1 compared to reflex Cr1, *t*_(20)_ = 4.323, *p* < 0.001; however, LVI was not significantly different between Cr2s for voluntary and reflex cough. The magnitude of change in LVT from Cr1 to Cr2 was significantly greater for voluntary cough compared to the change for reflex cough, *t*_(20)_ = 3.782, *p* = 0.001.

**Figure 4:**
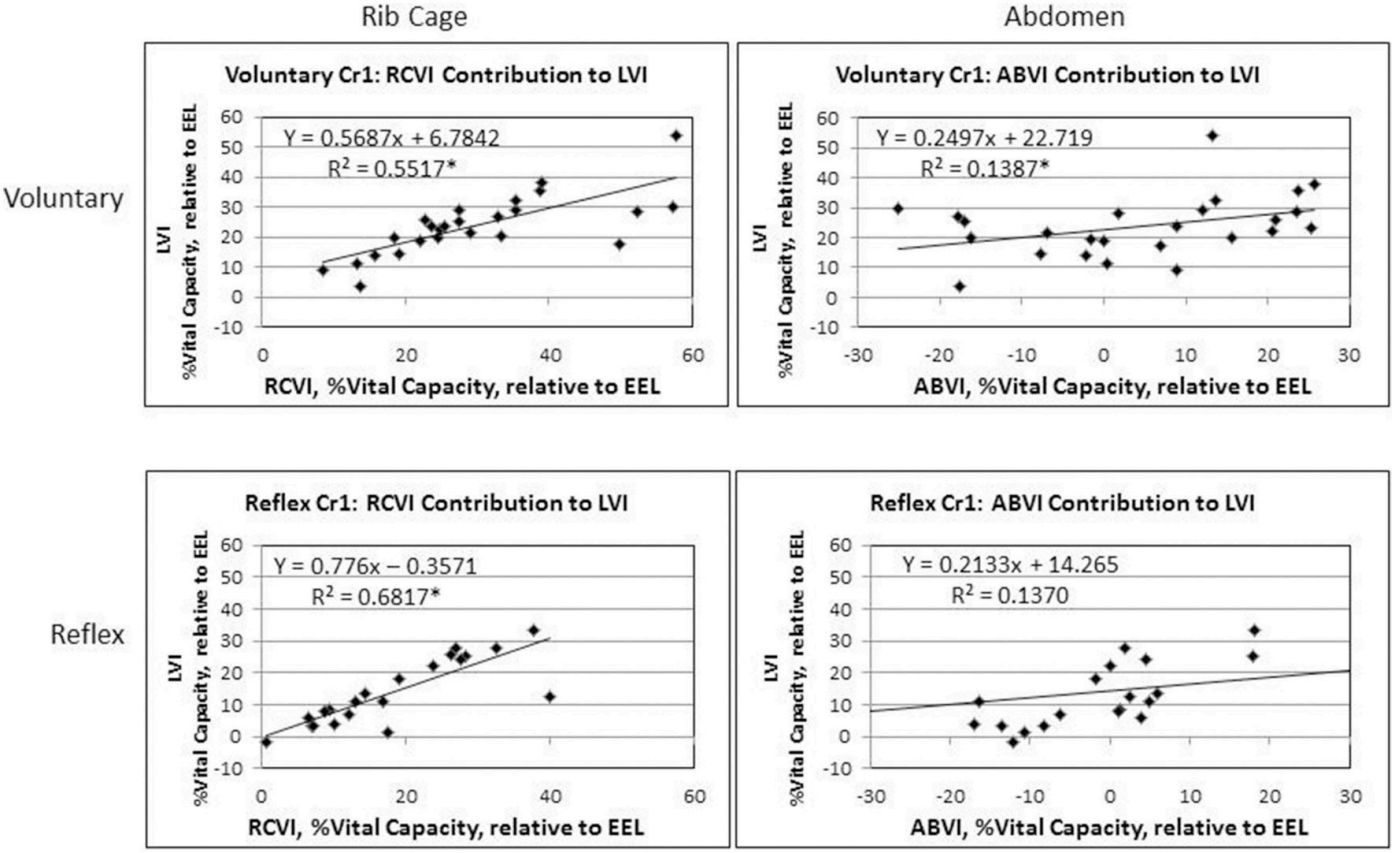
**Initiation measurements for reflex and voluntary cough: mean percent vital capacity relative to end-expiratory level (EEL) for the first cough responses**. RCVI and ABVI significantly contributed to LVI for voluntary cough; however, only RCVI significantly contributed to LVI for reflex cough. Lung volume initiation (LVI); rib cage volume initiation (RCVI); abdominal volume initiation (ABVI). ^*^Significant contribution to LVI (*p* < 0.05).

**Figure 5:**
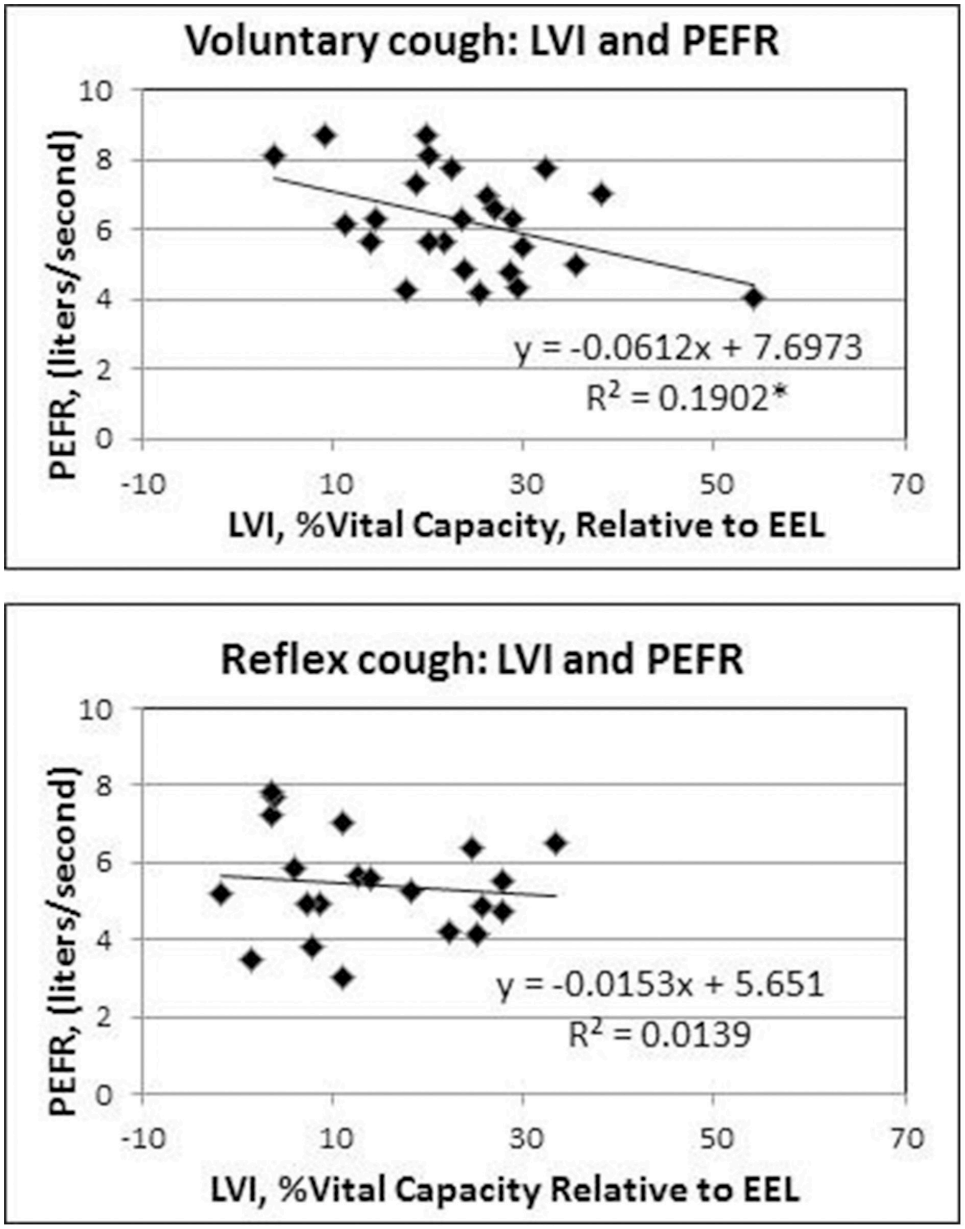
**Influence of lung volume Initiation (LVI) on peak expiratory flow rate (PEFR) for reflex and voluntary cough: mean percent vital capacity relative to end-expiratory level (EEL) for the first cough responses**. LVI significantly influenced PEFR for voluntary cough; however, LVI did not significantly influence PEFR for reflex cough. ^*^Significant contribution of LVI to PEFR (*p* < 0.05).

Results of the regression model showed that for voluntary cough, both RCVI [β = 0.751, *t*_(22)_ = 6.450, *p* < 0.001] and ABVI [β = 0.388, *t*_(22)_ = 3.332, *p* = 0.003] significantly influenced LVI [*R*^2^ = 0.702, *F*_(2, 22)_ = 25.923, *p* < 0.001]. For reflex cough, only RCVI [β = 0.785, *t*_(18)_ = 5.796, *p* < 0.001] significantly influenced LVI [*R*^2^ = 0.699, *F*_(2, 18)_ = 20.892, *p* < 0.001; Figure 4]. Finally, LVI significantly influenced PEFR [*R*^2^ = 0.190, *F*_(1, 23)_ = 5.401, *p* = 0.029] for voluntary cough but not reflex cough [*R*^2^ = 0.014, *F*_(1, 19)_ = 0.269, *p* = 0.610; Figure 5].

Intra-and inter-rater reliability analyses were completed on 20% of the data to ensure the accuracy and consistency of data analyses and measurement. The results revealed that measurement of the data was reliable (intra-class correlations Cronbach's Alpha = 0.948; inter-class correlations Cronbach's Alpha = 0.964).

## Discussion

This is the first study to examine lung volume, respiratory kinematic, and airflow parameters for both reflex and voluntary cough. The results revealed significant differences between the cough types that included greater LVI and LVE for voluntary cough compared to reflex cough, and differences in the kinematic strategies employed to achieve LVI. Specifically, the rib cage and abdomen (RCVI and ABVI) significantly influenced LVI during voluntary cough; however, only the rib cage (RCVI) significantly influenced LVI for reflex cough. In terms of cough airflow, peak expiratory flow rate (PEFR) was significantly higher for voluntary compared to reflex cough. However, there were significant differences in the contribution of LVI on PEFR between the cough types such that LVI influenced PEFR for voluntary cough, but not reflex cough. These data extend our understanding regarding the respiratory strategies utilized by healthy young adults to produce reflex and voluntary coughs.

Previous studies have shown that individuals modify kinematic strategies during various respiratory-related tasks based on multiple factors (Dromey and Ramig, 1998; Huber et al., 2005; Huber, 2007; Wheeler Hegland et al., 2009, 2011). For example, when healthy adults are cued to speak at increased loudness levels, they modify kinematic strategies by: (a) increasing LVI and RCVI compared to conversational speech to capitalize on higher elastic recoil forces of the lung, (b) reducing ABVI secondary to increased abdominal muscular tension and pressure, or (c) utilizing a combination of these two strategies (Huber et al., 2005). Our results indicate that cough, like speech, leads to modification of respiratory kinematics based on the task (voluntary vs. reflex cough).

The LVI of voluntary cough was significantly higher than that of reflex cough; however, for both cough types the rib cage was the main contributor to lung volume. In the upright position, most individuals are “thoracic breathers,” exhibiting higher rib cage volume compared to abdominal volume during tidal ventilation (Sharp et al., 1975; Hixon et al., 1976; Hoit, 1995). Further, rapid inspiratory and expiratory ventilatory maneuvers are largely accomplished by displacement of the rib cage secondary to the fast contractile speed of the rib cage musculature (Sharp et al., 1975; Farkas et al., 1985). These characteristics may have allowed the rib cage to respond more quickly during both types of cough in this study. Thus, the quick, ballistic quality of thoracic muscle contraction during cough may have contributed to this finding.

In contrast, abdominal behavior was highly variable and only significantly contributed to LVI for voluntary cough. The differential influence of the abdomen on LVI between the cough types may reflect physiological differences between the reflex and voluntary cough. Researchers have shown that reflex cough results in simultaneous expiratory and accessory inspiratory muscle (i.e., trapezius, pectoralis major, deltoid, latissimus dorsi) activation, increased mean abdominal EMG activation overall, decreased muscular burst duration, longer glottal closure, and increased intra-abdominal pressure compared to voluntary cough (Lasserson et al., 2006). On the other hand, voluntary cough results in coordinated activation of the expiratory muscles with a graded increase in EMG muscular activation and duration (Lasserson et al., 2006). Stephens et al (Stephens et al., 2003) identified increased intra-abdominal pressure and increased superior displacement of the diaphragm during reflex cough that was not observed during voluntary cough. Given that diaphragm contraction drives outward displacement of the abdomen during inspiration, the reduced inferior diaphragmatic displacement during reflex cough may have limited the contribution of the abdomen. Taken together, these findings suggest that the increased intra-abdominal pressure, co-contractions of the respiratory muscles, and muscular tension during induction of reflex cough limited the displacement of the abdomen prior to cough. For speech function, the characteristic of increased intra-abdominal pressure is associated with increased displacement of the rib cage upward and outward in the inspiratory direction (Hoit, 1995). It is therefore possible that the increased intra-abdominal pressure, and muscular tension that occurs during reflex cough increased reliance on RCVI, limited the contribution of ABVI, and ultimately reduced LVI compared to voluntary cough. These physiological and kinematic differences for reflex cough may reflect a biological protective mechanism to inhibit inhalation (i.e., reduced RCVI and LVI) prior to the cough event and prevent continued inhalation of irritants into the lower airways.

Interestingly, LVI influenced PEFR for voluntary, but not for reflex cough. Smith et al. (2012) previously identified that higher LVIs were associated with higher PEFRs for voluntary cough, and therefore, we hypothesized that this finding would be present for reflex cough as well. However, our hypothesis was not supported for reflex cough, suggesting that other factors within the respiratory and laryngeal subsystems influence PEFR more than changes in lung volume. During cough, the larynx plays a critical role in inspiration, narrowing the glottis, increasing laryngeal resistance, developing subglottal tracheal pressure, and allowing for high expiratory airflow from the lungs. Poliacek et al. (2003) found that the laryngeal adductors and abductors exhibited varying degrees of overlapping muscular activation during different types of induced reflex cough in a cat model. Co-contraction between laryngeal adductor and abductor muscles during reflex cough would alter the natural open glottal configuration of the larynx during inspiration to increase laryngeal resistance and reduce LVI. Further, co-contraction of the adductors and abductors during the expulsive phase may have limited volume and airflow through the glottis, effectively limiting the PEFR regardless of respiratory contribution.

### Potential implications for neural control

It is well established that the neural substrates controlling the initiation of reflex and voluntary cough differ. Voluntary cough is solely dependent upon cortical activation. Whereas, reflex cough requires peripheral stimulation, activation of the brainstem respiratory network, and activation of various cortical areas (some distinct from those activated for voluntary cough) to allow for modulation of the motor behavior (see Troche et al., 2014a for a more in-depth review). Although not explicitly tested in this study, the observed respiratory kinematic and airflow differences between the cough types may reflect differences in the neural control of each behavior.

For example, one of the most salient differences between reflex and voluntary cough is the presence of a sensory stimulus driving the behavior. The presence of a strong tussigenic stimulus may have inhibited the ability to achieve maximal LVI, possibly in relation to a strong urge-to-cough (UTC; Smith et al., 2012). The UTC is a respiratory sensation that precedes the reflex cough, and provides awareness of the tussigenic stimuli (Davenport et al., 2002; Davenport, 2009). As the UTC increases, the number of coughs produced increases (Vovk et al., 2007), indicating that the intensity of a cough-inducing stimulus may regulate the airflow characteristics of coughs produced. Additionally, it may be the magnitude of the UTC that also regulates inspiratory characteristics (Hegland et al., 2013), leading to the reduced LVI and differential contribution of the rib cage and abdomen for the reflex cough task in this study. This corroborates the findings of Smith et al. (2012) who identified that healthy young participants could not achieve targeted LVIs in the presence of a tussigenic stimuli (citric acid). Additionally, Hegland et al. (2013) found that the volume of air inspired prior to a reflex cough induced with capsaicin was not greater than the volume of air inspired during tidal breathing. In fact, lung volumes below EEL were frequently used prior to reflex cough (Hegland et al., 2013). Thus, the method for elicitation of cough, whether reflex or voluntary, results not only in neural differences, but kinematic and airflow differences as well. These findings are important given that voluntary cough is frequently used clinically to assess the effectiveness of both reflex and voluntary cough mechanisms. It is clear from the results of this study that voluntary cough motor output overestimates reflex cough motor output in healthy young adults. Therefore, separate evaluations of both voluntary and reflex cough may be necessary to more accurately assess cough as a mechanism of airway protection.

### Limitations and future directions

This study provides essential information related to the mechanical and airflow differences between reflex and voluntary cough; however, it is not without limitations. Previous studies have identified 200 μM capsaicin as a suprathreshold stimulus at which healthy young adults reliably cough in response (Vovk et al., 2007), but 4/25 (16%) of participants in this study did not respond to this stimulus. To ensure the collection of a reflex cough sensitivity threshold, future investigations will incorporate at least one concentration above 200 μM. Additionally, our future investigations will include multiple concentrations of capsaicin, with concurrent ratings of UTC, in order to better understand the relationship between the UTC and cough dynamics. Future investigations will target voluntary cough LVI and PEFRs that are similar to reflex cough production to determine if the respiratory kinematic differences exist at comparable lung volume and airflow rates. Given the likely role of the larynx for the determination of cough PEFR, future research should incorporate laryngeal measurements to identify features that may mediate cough effectiveness. Intra-abdominal pressure and respiratory muscle activity differences were not evaluated in this study and may help explain variability in abdominal contribution to LVI in future studies. It is also possible that our study was underpowered to adequately evaluate abdominal contribution to cough kinematics. Further, although this study successfully identified the relationship between the relative contribution of the rib cage and abdomen to LVI for both reflex and voluntary cough, the regression models used did not provide direct comparison of the beta weights and thus, limit the differences that can be inferred between the cough types.

## Conclusions

The results of this prospective study increase our understanding of the mechanistic and airflow differences between voluntary and reflex cough in healthy young adults. These differences likely resulted from a combination of increased respiratory and laryngeal muscle co-contraction, along with possible inhibition of inspiration secondary to a strong UTC that is associated with reflex cough as opposed to voluntary cough. Functionally, increased respiratory and laryngeal co-contractions and a strong perception of the UTC for reflex cough may serve to inhibit inhalation of a tussigenic stimulus in order to prevent further intake of foreign material into the lower airways. Knowledge of how volume is partitioned among the various parts of the respiratory apparatus during cough is fundamental to the understanding of cough function in healthy young adults and will serve as a means to compare the cough mechanisms in healthy older adults and in persons with disease. These findings are highly important to consider in patient populations with dystussia (cough dysfunction), such as Parkinson's disease, where the UTC is blunted (Troche et al., 2014b), and dyscoordination/co-contraction of antagonistic muscles are known physiologic changes (Hovestadt et al., 1989). The long-term aim of this research is to facilitate the development of treatment paradigms for cough dysfunction that can be utilized in patient populations with dystussia and airway compromise. We hypothesize that adjustments to lung volume and kinematic strategies during voluntary cough as part of an exercise model will contribute to the modification of the reflex cough motor function. For example, research has shown that healthy young adults can volitionally modify capsaicin induced reflex cough based on experimenter instructions (Hegland et al., 2012). As such, in future research studies we will evaluate the ability of healthy older adults and individuals with neurodegenerative disease to volitionally modify kinematic parameters (namely RCVI and LVI) to improve measures of cough airflow and effectiveness. These studies are particularly necessary given that there are currently no treatments available for people with down-regulated cough and dystussia.

## Author contributions

AB, MT, and KH conception and design of research; AB and KH acquired data and performed experiments; AB and JH analyzed data; AB, MT, KH, and JH interpreted results of the experiment; AB prepared figures; AB drafted the manuscript; AB, MT, JH, and KH edited and revised the manuscript.

## Grants

Dr. Troche's research is funded in part by a National Institutes of Health (National Center for Advancing Translational Sciences) Clinical and Translational Science Award through the University of Florida (UL1TR000064 and KL2TR000065).

Dr. Huber's research is funded in part by a National Institutes of Health R01 grant (R01DC9409), a grant from the Indiana Clinical and Translational Sciences Institute (CTSI, grant number TR000006) funded by the National Institutes of Health, National Center for Advancing Translational Sciences, Clinical and Translational Sciences Award, and funding from corporate sponsors.

Dr. Hegland's research is funded in part by the American Heart Association and BAE defense systems.

### Conflict of interest statement

The authors declare that the research was conducted in the absence of any commercial or financial relationships that could be construed as a potential conflict of interest.

## Abbreviations

LVI: Lung volume initiation
LVT: Lung volume termination
LVE: Lung volume excursion
RCVI: Rib cage volume initiation
ABVI: Abdominal volume initiation
PEFR: Peak expiratory airflow rate
Cr1: First cough response in a cough epoch
Cr2: Second cough response in a cough epoch.
